# Health Tract No. 331

**Published:** 1868-06

**Authors:** 


HEALTH TRACT No. 331

### LIVING BY RULE

As if a Medo-Persian Law, inflexible, is very unwise, especially if a person is in reasonable health. We have given a great multitude of counsels on the subject of health and disease, and in connection with the statement that we have not lost an hour from our office, on account of sickness in a quarter of a century and more, many have inquired with a good deal of interest "Do you live up to the rules you give others." Certainly not ; man is not a machine, that must be turned in a certain direction or it will be destroyed ; nor like a locomotive which must run on one fixed track, or not run at all. The Architect of all worlds made us for acting under a great variety of circumstances, and in infinite wisdom and benevolence has given to man a mechanism of wonderful adaptability, by which he can live healthfully on land or sea ; in the valley or on the mountain top ; in the tropics or at the poles ; on the barren rock or in the rich savannas. Our modes of life must be adapted to our age, our occupation and the peculiarities of our constitution. There are certain general principles which are applicable to all. Every man should be regular in his habits of eating ; should have all the sound sleep which nature will take ; should be in the open air an hour or two every day. when practicable, and should have a pleasurable and an encouragingly remunerative occupation, which keeps him a little pushed, and they are happiest who are in this last catagory ; at the same time, if a man accustoms himself to go to bed at nine o'clock, he need not break his neck, or get into a stew, if circumstances occur to keep him up an hour or two later, now and then ; and so with eating, exercise and many other things. No one ought to make himself a galley slave to any observance ; occasional deviations from all habits are actually beneficial ; they impart a pliability to the constitution, give it a greater range of healthful action. Don't go into a fit, if dinner is not ready at the instant. Deliver us from a machine man, a routinist, "for which we ever pray."

The following is continued from the March number :

knowledge which it collects; for in rescuing from untimely death the assembled children of poverty, science learns, as it could in no other way do, methods which enable it to rescue the children of wealth.

The more hopeful character of the most modern science had been in great part anticipated by the brave intellect of Andrew Combe. Before his time, it was too generally, if not universally assumed, that the symptoms of Consumption were a death-warrant; he proclaimed the reverse truth, and established it. He became in his own person the teacher and exemplar, both to physician and patient; and in his compact popular volume and regimen, he has recorded, in a form accessible to all, the conclusions of his practical experience. He did away many of the old coddling notions, which helped to kill the patient by stifling the pores of the skin, filling the lungs with bad air, softening the muscular system with inaction, and deadening the vital functions; a service scarcely more useful in reconciling the patient to the restorative influences of nature, than in returning hope to the afflicted relatives, and in showing what might be done by common sense and diligence. At an early age, Andrew Combe was found to be in a Consumption—words which were formerly accepted as a death-warrant, in submission to which the awed patient duly laid down and died; Andrew Combe lived more than twenty years longer, a life of activity, usefulness, and temperate enjoyment.

"The 'People's Journal,' for July, one of the most popular European publications, has an interesting article in relation to the Consumption Hospital, founded at Brompton; and few institutions have risen so rapidly. It has a long list of noble and wealthy subscribers, with the Queen and most of the royal family at its head. 'As death has abundantly proved the mortality of the disease, so, paradoxical as it may seem, death also supplies us with evidence that the chief structural lesions of Consumption, tubercles in the lungs, are not necessarily fatal. The writer of these lines can state, from his own observation, (which has not been limited, and is confirmed by that of others,) that, in the lungs of nearly one-half of the adult persons examined after death from other diseases, and even from accidents, a few tubercles, or some unequivocal traces of them are to be found. In these cases, the seeds of the malady were present, but were dormant, waiting for circumstances capable of exciting them into activity, and if such circumstances could not occur, the tubercles gradually dwindled away, or were in a state of comparative, harmless quiescence. This fact, supported by others, too technical to be adduced here, goes far to prove an important proposition, that Consumptive disease is fatal by its degree, rather than by its kind; and the smaller degrees of the disease, if withdrawn from the circumstances favorable to its increase, may be retarded, arrested, or even permanently cured. There are few practitioners of experience who cannot narrate cases of supposed Consumption which, after exhibiting during months and even years, undoubted symptoms of the disease, have astonished all by their subsequent, more or less, complete recovery. Cautious medical men have concluded themselves mistaken, and that the disease was not truly tuberculous; but, in these days, when the detection and distinction of diseases is brought to a perfection bordering on certainty, the conclusion that recoveries do take place from limited degrees of tubercles of the lungs, is admitted by the best authorities, and is in exact accordance with the above-mentioned results of cadaveric inspection. Consider properly, and you will be ready to admit the truth of what has been already established by experience, that Consumption may be often prevented, arrested or retarded by opportune aid. On this point we know that many medical men are utterly incredulous, and stigmatize others who are less so, in no measured terms; but, with the present rapid improvements in all the departments of medical knowledge, there is less ground for such incredulity than there was for that which opposed and ridiculed Jenner in his advocacy of vaccination as the preventive of small-pox.'

In view of the above and other testimonials of the most distinguished living writers in favor of the curability of Consumption, it is impossible for any well-informed and well-balanced mind any longer to deny it. We cannot conceive it possible that so many great men should be so much deceived on a point which they have made it the business of a life-time to investigate and study.

"SUICIDE BY STARVATION.

"A very curious example of suicide by means of starvation occurred some years ago in Corsica. During the elections, the Sieur V. rushed into the electoral college armed with a dagger, which he plunged into the breast of a man who had done him some injury. The man fell dead at his feet. The assassination was committed in the full light of day, and in the presence of an assembled multitude.

"V. was tried, found guilty, and condemned to death. His high spirit and resolute character were well known, and it was suspected that he would seek, by a voluntary death, to evade the disgrace of perishing on the scaffold. He was therefore vigilantly watched, and every precaution taken to deprive him of the means of putting an end to his existence.

"He resolved to starve himself to death during the interval which elapsed between the sentence of the Court or Assizes and the reply which the Court of Cassation would make to the appeal he had addressed to it.

"He had succeeded in concealing from the observation of his jailers a portion of the food with which they supplied him, so as to make it be believed that he regularly took his meals. After three days' abstinence, the pangs of hunger became insupportable. It then suddenly occurred to him that he might the more speedily accomplish the object he had in view by eating with avidity. He thought that the state of exhaustion to which he was reduced would unfit him to bear the sudden excess, and that it would inevitably occasion the death he so ardently desired. He accordingly sat down to the food which he had laid aside, and ate voraciously, choosing in preference the heaviest things. The consequence was that he was seized with a violent fit of indigestion, from which, contrary to his expectation, the prison doctor speedily cured him.

"He then resumed his fatal design. He suffered again what he had undergone before. The torture was almost beyond his strength. His thirst, too, was intolerable. It overcame his resolution. He extended his hand towards the jug of water which had been placed in his cell. He drank with avidity, and, to use his own expression, *was restored to life.*

"To avoid yielding again to a similar temptation, he daily took the precaution of overturning the jug of water which was brought to him. Lest he should be induced to raise it to his lips, he threw it down with his foot, not venturing to touch it with his hand. In this manner he passed eighteen days.

"Every day, at different intervals, he noted down in his album a minute account of his sensations. He counted the beatings of his pulse, and marked their number from hour to hour, measuring with the most scrupulous attention the gradual wasting of his strength. In several parts of his melancholy *memento*, he declares that he felt it harder to bear the agonies of thirst than those of hunger. He confesses that he was frequently on the point of yielding to the desire of drinking. He nevertheless resisted.

"He was surprised to find his sight become more and more clear, strong, and accurate; it appeared to him like the development of a new sense. The nearer he approached his latter moments, the more his power of vision seemed to increase. On this subject he thus expresses himself: 'It appears as though I could see *through* the thickest walls.' His sense of feeling likewise attained the most exquisite sensibility. His hearing and smelling improved in a similar degree. His album contains many curious statements on these subjects.

"The Sieur V. had devoted some attention to anatomy and physiology; and he attributes the increased acuteness of his senses to the way in which the intestinal irritation acted on the nervous system.

"His ideas, he says, were numerous and clear, and very different from anything he had experienced in moments of excitement or intoxication. They were all directed to logical investigation, whether he applied them to an analysis of material objects, or to philosophic contemplation. He also felt himself inspired with a singular aptitude for mathematical calculation, a study for which he had previously felt very little inclination. In short, he declares that he never derived so much gratification from his intellectual condition, as throughout the whole duration of his physical torture.

"He made notes in his album to the last moments of his existence. He had scarcely strength sufficient to hold the pencil with which he traced the following words: 'My pulse has nearly ceased to beat—but my brain retains a degree of vigor which, in my sad condition, is the greatest solace Providence could bestow on me. It is impossible that I can live out this day. My jailers watch me, and fancy they have adopted every precaution. They little think that I have outwitted them. Death annuls the sentence which has been pronounced on me. In another hour, perhaps, they will find nothing but a cold corpse.'

"V. expired as he foretold. His album has been carefully preserved. It is a record replete with interest to medical professors. The slow torture, endured with so much courage, and described with such remarkable clearness, renders it one of the most curious documents in the annals of medical science."

Illustrating the same point, a gentleman, Mr. I. F. H., stated to the author that he was once under medical treatment for some affection of the eyes, requiring a very scanty diet. His general health was excellent, but he was always hungry; yet so far from having any sense of debility, he had, when he went out into the street, an elasticity of mind and body, an instinctive desire of locomotion, which caused him to feel as if he could almost fly, and a joyousness of spirit, which was perfectly delightful.

These two cases strikingly show, that with a smaller amount of food, and consequently of blood, men are cheerful in mind and active in body ; ☞ therefore, a small amount of food, perfectly digested, gives more health and strength than a larger, not so. It is better, incomparably better, to feel a little hungry all the time, than to feel full, oppressed, heavy, with over eating.

Every patient of mine, who ever expects to get well, must keep this fact constantly and practically in view. It is too much the custom to measure one's health by the avidity of his appetite and his increase in flesh, as if he were a pig; forgetting that a voracious appetite and fat are always indications of a diseased body. A uniform moderate appetite is the attendant of good health. A racer's ribs must be seen before he is fit for the track, because then he is most capable of endurance.

The next incident shows, that with a moderate amount of substantial food and cold water, such being prisoner's fare, men may live for many years, with but little exercise, in the dark vaults of a prison, breathing all the time an atmosphere not very pure, as may be readily supposed. And it is earnestly hoped that the incidents narrated will leave upon the mind of every reader a life-long impression as to the value, both to the sick and the healthy, of living habitually on a moderate allowance of plain, substantial, nourishing food. It may be well to recollect here that it is not the quality, so much as the quantity of food, which lays the foundation every year of innumerable diseases and deaths. Let it be remembered, also, that men need a variety of food ; living on one or two kinds for a length of time will always undermine a healthy constitution. Milk only has all the elements of life ; and any other one kind of aliment, used indefinitely as to time, will as certainly deteriorate the constitution, bodily and mental, as anything that is planted will deteriorate if kept for successive years in the same field unrenewed. The popular notion that one or two kinds of food at a meal is *most wholesome*, is wholly untrue. On the contrary, several kinds at a meal, other things being equal, are more conducive to our well-being. Quantity, and not quality, is the measure of health.

### COUNT CONFALIONERI

wrote from the great jail of Vienna as follows :—

"I am an old man now, yet by fifteen years my soul is younger than my body : fifteen years I existed, for I did not live. It was not life in the self-same dungeon, ten feet square. During six years I had a companion ; nine years I was alone. I never could rightly distinguish the face of him who shared my captivity in the eternal twilight of our cell.

"The first year we talked incessantly together. We related our past lives, our joys forever gone, over and over again.

"The next year we communicated to each other our ideas on all subjects.

"The third year we had no ideas to communicate ; we were beginning to lose the power of reflection.

"The fourth, at intervals of a month or so we would open our lips, to ask each other if it were indeed possible that the world were as gay and bustling as it was when we formed a portion of mankind.

"The fifth year we were silent.

"The sixth, he was taken away, I never knew where, to execution or to liberty. But I was glad when he was gone: even solitude was better than that pale and vacant face. After that, I was alone.

"Only one event broke in upon my nine years' vacancy. One day, it must have been a year or two after my companion left me, my dungeon door was opened, and a voice, I knew not whence, uttered these words : 'By order of his Imperial Majesty, I intimate to you, that one year ago your wife died.' Then the door was shut. I heard no more. They had but flung this great agony in upon me, and left me alone with it again."—Phil. Pennsylvanian, March 2, 1850.

Having shown the bearing which food has on health, I desire to make some statements as to the value of air and exercise in the same direction. These will be given succinctly, in the hope that the intelligent reader will study them and apply them at length, especially if he should come to me for medical advice. My habit is not merely to cure when I can the patient who comes to me, but to induce him to study and understand his own case and constitution, so that by the application of general principles he may afterwards be able to regulate his health under all ordinary circumstances, as far as it can be done by diet, air, exercise, and regularity of personal habits ; but never venturing to take an atom of medicine, however simple, except by the special advice of an educated, experienced physician.

### IMPORTANCE OF PURE AIR TO HEALTH.

Men are reported to have lived three weeks without food, but without air we cannot live three minutes. The lungs of a full-sized man weigh about three pounds, and will hold twelve pints of air; but nine pints are as much as can be inhaled at one full breath, there being always a residuum in the lungs ; that is, *all* the air that is within them can never be expelled at once. In common, easy breathing, in repose, we inhale one pint. Singers take in from five to seven pints at a single breath. We breathe, in health, about eighteen times in a minute ; that is, take in eighteen pints of air in one minute of time, or three thousand gallons in twenty-four hours.

On the other hand, the quantity of blood in a common-sized man is twenty pints. The heart beats seventy times in a minute, and at each beat throws out four tablespoons ; that is, two ounces of blood : therefore, there passes through the heart, and from it through the lungs, an amount of blood every twenty-four hours equal to two thousand gallons.

The process of human life, therefore, consists in there meeting together in the lungs, every twenty-four hours, two thousand gallons of blood and three thousand gallons of air. Good health requires this absolutely, and cannot be long maintained with less than the full amount of each ; for such are the proportions that nature has ordained and called for. It is easy, then, to perceive, that in proportion as a person is consuming daily less air than is natural, in such proportion is a decline of health rapid and inevitable. To know, then, how much air a man does habitually consume, is second in importance, in determining his true condition, to no other fact; is a symptom to be noticed and measured in every case of disease, most especiallly of disease of the lungs; and no man can safely say that the lungs are sound and well and working fully, until he has ascertained, by actual mathematical measurement, their capacity of action at the time of the examination. All else is indefinite, dark conjecture. And I claim for myself to have been the first physician in America who made the measured amount of consumed air an essential element as to symptoms, in ascertaining the condition of persons in reference to the existence of Consumptive disease, and making a publication thereupon. The great and most satisfacfactory deduction in all cases being this, that if, upon a proper examination, the lungs of any given person are working freely and fully, according to the figures of the case, one thing is incontrovertibly true, demonstrably true, that whatever thousand other things may be the matter with the man, he certainly has nothing like Consumption. And Consumption being considered a fatal disease by most persons, there is quite a willingness to have anything else; and the announcement and certainty that it is not Consumption, brings with it a satisfaction, a gladness of relief, that cannot be measured.

On the other hand, just in proportion as a person is habitually breathing less air than he ought to do, in such proportion he is falling fast and surely into a fatal disease. This *tendency* to Consumption can be usually discovered years in advance of the actual occurrence of the disease; and were it possible to induce the parents of children over fifteen years of age to have investigations as to this point in the *first place*, and *then* to take active, prompt, and persevering measures to correct the difficulty, and not one case in a thousand need fail of such correction, with but little, if any medicine, in most instances many, many a child would be prevented from falling into a premature grave, and would live to be a happiness and honor to the old age of those who bore them. Persons who live in cities and large towns think, and wisely so, that the teeth of their children should be carefully examined by a good dentist once or twice a year; but to have the condition of the lungs examined, and, if need be, rectified, who ever thought of such a thing? And yet, as to practical importance, it immeasurably exceeds that of attention to the teeth. The latter are cared for as a matter of personal appearance and comfort; the lungs are a matter of life and death. We can live and be happy without a tooth, but without lungs we must prematurely die. Were the condition of the lungs, after such an examination as I have suggested, a matter of opinion or conjecture only, I would not propose it; but it is not: it is a thing of numerical measurement, of mathematical demonstration, as to the one point, Do the lungs work freely and fully or not? If they do not, declining health is inevitable, sooner or later, unless their activity is restored, which, however, can be done in the vast majority of cases.

### YOUNG PERSONS.

While speaking of the health and habits of the young, it may be well further to state, that wrong indulgences debilitate the system; in time, the mind becomes unable to fix itself upon any subject profitably. Exhausting discharges further weaken the energies, and idiocy sometimes supervenes, in various forms and degrees of epilepsy; at other times, fatal symptoms of Throat-Ail and Bronchitis. (See Trousseau and Belloc.)

### A CASE.

"A youth, aged nineteen, indulged freely for some time, and at length began to experience pains about the throat. The voice was altered; shrill at first, then entirely lost. Swallowing liquids became impossible. He spit up large quantities of matter, and died after a year's illness. The lungs, on examination, were entirely sound, but the whole throat was ulcerated."

Throat-Ail and Consumption are diseases of debility, and it may be easily supposed that no progress can be made towards a cure while causes of debility *are in operation*. This statement is made here to save the necessity, in all cases, of more direct inquiries. If, however, there is no personal control, parents may apply for their children, and permanent relief be obtained without wounding the feelings or self-respect of the ailing party, who indeed may be blameless.

### MISCELLANEOUS CASES.

(851. Sept. 2.) Your lungs are unimpaired; they are in full working order. There is no tendency at this time to Consumptive disease. Your ailment is dyspeptic laryngitis, complicated with a slight pleuritic affection, and with proper attention you will get well. At the same time, it is important for you to know, that these throat affections are among the most incurable of all diseases when once fully established. This consideration should induce you to commence *at once* a proper course of treatment, and to *persevere in it* until you are *perfectly* restored to health.

*Note.*—His principal ailment was an uneasy feeling in the throat, a frequent clearing of it, and an almost constant pain in the left breast. He wrote me in three weeks, that my prescriptions were acting admirably, and that he was getting well.

(852. Sep. 2.) Your ailment is common tubercular disease, mainly tending to fix itself on the lungs, and next on the bowels. Decay of the lungs has not yet begun to take place; they are becoming inactive, about one-tenth of them doing you no efficient good. There is a reasonable probability that the disease may be arrested at this stage. A return to good health is by no means impossible; it is doubtful. The throat ailment is nothing more than what may arise from a dyspeptic condition of the stomach, liable to end in tubercular ulceration in your case, your lungs being already tuberculated to some extent; the right side slightly more than the other.

*Note.*—He complained chiefly of spitting blood, cough and debility; had been using cod liver oil for several months to no purpose. I have not heard from him since giving the opinion.

(853. Sept. 2.) You have chronic laryngitis, torpid liver, lungs acting imperfectly. There is no decaying process, no Consumptive disease, and I see no special reason why you may not, with judicious treatment, recover your health.

He complained chiefly of husky voice (had to abandon preaching), constipation, and variable appetite. In five months he wrote me that he "was able to enter upon his pastoral duties," and had been discharging them three months.

(854. Sept. 12.) Your lungs are not in a safe condition; one-third of them are now useless to you. It will be necessary for you to use diligent efforts to arrest the progress of your disease, and spare no pains in doing so.

*Note.*—Complains chiefly of spitting blood, cough, sore throat, debility. He appears to be getting well rapidly.

(855. Sept. 7.) Your disease is common consumption of the lungs; one fourth of them are doing you no good; a part of them are irrecoverably gone; therefore, under no circumstances can you be as stout and strong as you once were. The decay of your lungs is progressing every hour. If that decay is not arrested, you cannot live until spring. Whether that decay can be arrested I cannot tell. It is possible that it may be done. It is not my opinion that it can be done.

*Note.*—Chief symptoms harassing cough, drenching night-sweats, daily expectoration of blood, constipation, irregular appetite, great emaciation and debility, could scarcely walk around one square. In three weeks he could walk twenty squares in a day without special fatigue. Here he ceased very unexpectedly to call upon me. Being a favorite child of his father, I took great interest in his case. Whether he suddenly relapsed and died, or thought he could get along now without farther aid from a physician, I do not know.

### A MERCHANT.

"At this time the lungs are untouched by disease; they do not work as free and full as they ought to do, but it is impossible that there should be any decay, or that they should be tuberculated to any extent. If your present weak state of health continues, the system will become so debilitated by winter, and so susceptible to impressions from cold, that you will in all probability fall into an eventual decline. At this time, nothing is the matter with you but symptoms arising from a torpid liver and impaired digestion. Your health can be certainly restored."

*Note.*—Aged thirty; he had spitting of blood, pains in the breast, and other symptoms which greatly alarmed himself and friends, as pointing to settled Consumption. He got perfectly well with little or no medicine, and remains so to this day.

On the same day, September 18, a young woman came for examination, having walked several squares.

*Opinion.*—"You are in the last stages of Consumption. A large portion of the lungs is utterly gone; the decay is rapidly progressing, and nothing can arrest it. Death is inevitable before the close of the year."

*Note.*—She had a hoarse, loud cough, cold feet, chills, no appetite, irregular bowels, difficult breathing on slight exercise. I did not prescribe. She died in a short time.

(714.) J. S., married, aged 40, an officer in the Mexican war, and severely wounded at Cerro Gordo, complained most of *cough, weakness, sweating at night*, and *shortness of breath.* Any sudden movement of the body or mental emotion produced almost entire prostration. Had lost one-ninth of his weight.

*Opinion.*—"Your lungs are in good working order, no decay, not an atom; the yellow matter expectorated is a morbid secretion from the windpipe and its branches. Your heart is affected; the calibre of its blood vessels is too small to transmit the blood with sufficient rapidity; hence the fluttering and great debility on any sudden motion or protracted exercise, for these but increase the quantity of blood to be conveyed away. Your ailments depend on constitutional causes to a great extent, and in proportion are capable of removal."

I heard of this gentleman no more for one year, when he came into my office a well man in every respect, saying that he began to get well in three days after taking the first weekly pill, and thought as he was doing so well, there was no necessity of writing.

A case (988) similar, in some respects, is now under treatment: great throbbing of heart and weakness on slight exercise; a violent beating in the temples the moment he lays his head on a pillow at night. This does not occur when he lies on his back. Frequent numbness and pricking sensation in left arm and leg; tosses and tumbles in bed for hours every night before he can get to sleep; great general weakness, and total inability to walk; riding in any kind of a carriage over a rough road, often but not always, brings on sick headache; has frequent distress at stomach; pulse one hundred; much dispirited, and has fallen away more than one-sixth.

*Opinion.*—"Your ailment is a symptomatic heart affection, depending now, mainly, on constitutional causes, originating in over efforts of mind and body. The lungs are sound and well."

In three weeks he writes, each of the two weekly pills brought away large quantities of stuff, yellow as yolk of egg, with masses of a colorless, stringy substance, and left my bowels regular. I now sleep as well as I could wish; very little pain in the side; stomach no longer distresses me. I have gained strength, but no flesh, and some throbbing yet remains.

*Note.*—This man will probably get well if he continues to follow the directions as well as at the beginning. He had been advised to exercise his arms and the muscles of his chest a great deal, and was told that he must work, and thinking he could accomplish both at the same time, and being naturally industrious, he began to saw wood for family use during the coming winter; but every day he became weaker and worse, until he could scarcely stand up. This being a heart affection, every moment of such exercise necessarily aggravated the malady.

This shows the mischievous effects of taking a wrong view of a case and of following the advice of every person one meets with. Many persons are advised to death. Over-confident advice is the attendant of inexperience and ignorance. It is forgotten that unpaid advisers, being well themselves, do not endanger their own lives, in case their recommendations are inefficient, if, indeed, not positively hurtful. Many are infatuated with *vegetable remedies*, taking it for granted that they can do no harm, even if they do no good; forgetting that in many cases a loss of time is equivalent to a loss of life, and that the most virulent poisons in all nature—those which produce almost instantaneous death—are of vegetable origin, such as nicotine, prussic acid, and the like.

I. Q. H., married, aged forty-eight; had a distressing cough, which, with a severe pain below the point of the right shoulder-blade, prevented any refreshing sleep. He arose every morning sweaty, haggard, and weary; no appetite, and daily expectoration of large quantities of matter. He had fallen off forty-two pounds, and was greatly depressed. I informed him that his lungs were not diseased, and that there was no necessary obstacle to his recovery. His friends thought he became worse under my treatment, for at the end of four weeks he was confined to his bed day and night, with frequent rigors and flushes. The pain steadily increased, at times aggravated almost beyond endurance by a cough, which I thought nothing could safely control, and hence gave nothing for it. He thought he could not live unless speedily relieved; his relative, a physician, came to remonstrate against my "holding out hopes of recovery to a man who was evidently sinking with Consumption." I informed the patient he was better; that he would probably need no more medicine, and explained to him the reasons for such an opinion. In a few days his strength began to increase, and he walked out. He left the city soon afterwards, and now, at the end of three years, he is a hearty, healthy man, weighing upwards of two hundred pounds, having taken no medicine since he saw me. I considered his case to be one of great torpidity of the liver, with abscess, and treated it accordingly. The reader may see by this, how important it is sometimes to know that a case is not Consumption, and also the value of a steady resistance against ignorant interferences.

(July 23.) "Your lungs are not diseased, nor are they even impaired in their action. There is not only no Consumption in your case, but there is a less tendency that way than in most persons. You have not merely lungs enough for the ordinary wants of the system, but a large amount in reserve. Your whole ailment is a dyspeptic condition, and there is no reason why a rational habit of life should not restore you to as good health as you have ever enjoyed, without any medicine whatever."

He complained of pain in the breast, large expectoration, voice sometimes husky, and a tightness across the chest.

(July 23.) "Your lungs at this time are not in a satisfactory condition, more than one-sixth of them being valueless to you. A portion at the top of the right breast has decayed away. Your case is one presenting all the ordinary symptoms of common Consumption. It will be altogether impossible for you to arrest the progress of your disease if you continue your present habits of business (printer). If you pursue an out-door calling, and acquire judicious habits of life, it is probable that your disease may be arrested, and that you may be restored to renewed health."

*Note.*—As he had a good appetite, was working daily at his trade, and did not feel very bad, he thought it not advisable to abandon his calling, and died in three months.

(Nov. 8.) "Your lungs are whole, sound, and in full working order. There is at present no appearance of Consumptive disease. Your ailments arise wholly from general constitutional causes, and may be removed by proper and rational habits of life and conduct."

*Note.*—He was not satisfied with my opinion; was fully impressed with a belief that he was falling into a decline, and insisted upon repeated examination. He was a man of wealth, of fortunate social relations, and very naturally dreaded death—too much so for a man. He observed faithfully the directions given, no medicine was advised, and wrote in three months that he was as well as he ever was in his life; his chief complaint was an "uneasy sensation about the heart," and some "trouble in the throat."

(Nov. 9.) "Your lungs are not diseased materially at this time. They do not work fully, but there is no decay. Your ailment is Chronic Laryngitis, of a very dangerous and aggravated character. It is very doubtful whether you will get well. Something may be done for you by a rigid attention to all the directions given."

*Note.*—He could not speak above a whisper; swallowed food with great difficulty and pain. He remained under the treatment of his family physician, and died in seven weeks."

(849.) "You are suffering under the combined influence of dyspepsia and consumptive disease, and they mutually aggravate each other. One-fifth of your lungs are now useless to you. This is a very serious deficiency. The extent to which you may be benefited, can only be ascertained by attention to directions given. Your case is not hopeless, yet it is critical and of a very grave character." He died in five weeks. He could not or would not control his appetite, and the author ceased to prescribe, as is his practice when instructions are not implicitly followed.

(Aug. 30.) "All your ailments arise from a want of natural proportion between exercise and eating. If these were properly regulated, you would get well without any other means, as the lungs are sound, healthy, and entire. You are too full of blood, and it is not healthful; hence it does not flow freely, but gathers about the internal organs, oppressing them and giving rise to any number of ailments, constantly varying as to character and locality. Make less blood and take more exercise, according to the printed instructions given you, and your return to good health will be speedy and permanent."

She complained of pains and oppressions, particularly about the chest, tickling cough, &c. I heard no more of her for six months, when her husband, a Southern planter, called to express his satisfaction, and to say that she was in good health, and had been for some time.

(Sep. 30.) "Your disease is common consumption of the lungs. It began at the top of the right breast, and after making some ravages there, it ceased and attacked the left, which is now in a state of continued decay. It may spontaneously cease on the left side, as it did on the right; in that event, life would be preserved for the present. Without such an occurrence as just named, one-half of the lungs being useless to you, the constitution usually fails in six or eight weeks, and sometimes much sooner." She died in six weeks.

Frail and feeble persons often outlive by half a life-time the robust and the strong, because they feel compelled to take care of themselves, that is, to observe the causes of all their ill-feelings, and habitually and strenuously avoid them. Our climate is changeable, and in proportion unhealthful. In New York City, for example, during one week in December last, in which the thermometer ranged from five degrees above Zero to fifty-five, there were forty-one deaths from inflammation of the lungs, while the ordinary number is about fifteen. The healthy disregard these changes to a great extent, and perish within a few days. The feeble are more sensitive to these changes; they increase their clothing and their bedding with the cold, and with equal care diminish both, with the amount eaten, as the weather grows warmer, and thus long outlive their hardier neighbors. These precautions, with others, must all observe, THROUGH LIFE, who have been cured of an affection of the throat or lungs. Let this never be forgotten, for the oftener you are re-attacked, the less recuperative energy is there in the system, and the less efficient will be the remedial means which once cured you, unless by months of continued attention and wise observances you give the parts a power and a strength they never had before. This can be done in many cases.

But once cured, avoid the causes which first injured you. If you put your hand in the fire, you may restore it, but however magical may be the remedy, that hand will be burned as often as it is placed in the fire, without any disparagement of the virtues of the restorative. No cure of your throat or lungs will render you invulnerable. What caused the disease in the first instance will continue to cause it as long as you are exposed to them. No promise is given you of permanence of cure longer than you are careful of your health. The safer plan by far will be to consider yourself peculiarly liable to the disease which once annoyed you, and make proportionate endeavors to guard yourself habitually against its advances. All assurances that any mode of cure will afford you a guarantee against subsequent attacks, are deceptive. No medicine that any man can take in health will protect him from disease. There is no greater falsity than this, that if you are well, a particular remedy, or drink, or medicine, will fortify the system against any specified disease, whether cholera, yellow fever, or any other malady. So far from this being so, it is precisely the reverse. Doubly so; you are thrown off your guard, and in addition you make the body more liable to the prevalent malady by poisoning the blood; for whatever is not wholesome food, is a poison to the system, pure water excepted. Nothing, therefore, will protect a healthy man from disease but a rational attention to diet, exercise, cleanliness, and a quiet mind; all else will but the more predispose him to it. But when once diseased and then cured, these things are not sufficient to keep him well; he must avoid what first made him an invalid, otherwise permanent health is not possible, but a speedy relapse and death are inevitable, as to Throat-Ail, Bronchitis, and Consumption.

### DANGER OF CUTTING TONSILS.

M. Landouville removed an enlarged tonsil of a woman, aged 21. In eight days she had uncontrollable spitting of blood, which was constant, besides vomiting a large quantity. Small pulse; extremities cold. The danger was imminent. Various means had already been adopted in vain; such as ice externally, styptics internally; then pressure with lint dipped in lemon juice; but it was at length controlled by pressing ice against the spot with forceps. (See *Hays' Med. Jour.*, October, 1851.) Other cases are given in medical publications; they are not of frequent occurrence, but each one operated upon is liable to experience disagreeable results. An operation is seldom necessary—not one case in twenty. And as in the case above, the danger was not over for a week after the operation had been performed, others who have the tonsils taken out have cause for a lengthened and most unpleasant suspense.

It must not be forgotten that Throat-Ail is in very many instances wholly unmanageable, and ends fatally, simply from its being thought lightly of, until it has produced such a state of general irritation throughout the system, that the constitutional stamina is exhausted, and the pulse is habitually a fourth, or third, or even more, above the natural standard. Most generally, such cases go on to a fatal termination, in spite of all modes of treatment. This is so uniformly the result, that any certain benefit in such cases cannot be promised, nor is it just that the general principles of treatment should suffer discredit from failure here; they are admirably and uniformly successful when ever they are applied in the early stages of the disease It is to invoke prompt attention to the first and earliest symptoms of Throat-Ail, that pains have been taken in these pages to describe them plainly, clearly, and distinctly.

### CELL DEVELOPMENT.

The human body is in constant transition. The particles of which its structure is constituted are not the same in position and relation for any two minutes in succession. Thousands of atoms which compose it the present instant are separated from it the next, to make a part of it no more; and other thousands, which are a portion of the reader's living self while scanning this line, will have been rendered useless and dead on reading the next. There are two different armies of workers, whose occupations cease not from the cradle to the grave. One army, composed of its countless millions, is building up the body; the other removes its waste; one party brings in the wood and the coal for the fire-place and the grate, the other carries away the ashes and the cinders;—the builders and the cleansers. When the builders work faster than the cleansers, a man becomes fat, and over-fat is a disease. When the cleansers are too active, the man becomes lean, and wastes away to a skeleton, as in Consumption. Health consists in the proper equilibrium of these workers.

Every movement of the body, every thought of the mind, is at the expense of a portion of the material frame; that is to say, certain atoms of the living body are killed by every action of the mind, by every motion of the body, and being dead, are useless. But they must be removed from the body, or these "heaps of slain" would fill up the workshop of life, and the whole machinery would stand still; the fire-place would be filled with ashes, the furnace clogged with cinders, and the grate be useless. Vast masses of these dead atoms are pushed, worked out, or thrown from the body at the surface. At any night, on undressing, the cleanliest person may rub from the body countless numbers of these dead atoms, a teaspoon-ful of them may be gathered from the feet at a single washing, if long neglected. Hence the value of thorough daily frictions to the skin, as promotive of health, because, on an average, we all eat about one-third more than is needed; thus throwing on the cleansers a third more labor every twenty-four hours than they were designed to perform. By the frictions we come to their aid artificially. They are wise who perform these frictions daily and well; but wiser they by far who do not eat the extra one-third, and consequently do not need to be scrubbed and bathed and washed every day of their existence, to save them from the effects of over-feeding. Better eat less and save trouble. The surplus third would feed half the poor of the land.

But a larger portion of these dead atoms are scattered in the more interior parts of the body, and the cleansers remove them by first rendering them fluid, as solid ice or snow is made fluid by heat. It is then, as it were, sucked up by these cleansers, and conveyed finally to the blood, just at the heart, where they are mingled together and sent direct to the lungs, where they meet with the pure air that is breathed. Here an exchange takes place between the air and the blood. The air gives to the blood its oxygen, its life, while the blood gives its death to the air. Hence it is that the air gives life as it goes into the lungs, but gives death if breathed unmixed as it comes from the lungs; that is, if a healthy person were to breathe for three minutes no other air than that which has just come out of the lungs of another man, in three minutes he would die. Hence my insisting so much on causing

Consumptive persons to breathe the largest possible amount of pure air; it unloads the blood more perfectly of its dead atoms, and also gives life to the essence of food which it also meets in the lungs; that is, puts the finishing work to its becoming living blood.

Let us notice next the builders, whose work is to supply new and living particles as fast as the old ones fall off and die. These new particles are in the blood, which delivers its living freight as it flows through the body, as a steamer delivers its freight to the thousand different ports as it ploughs along the majestic Mississippi. Whenever a living particle comes to the point where it is needed to supply the place of one just fallen or dead, by some inscrutable, inexplicable agency, as quick as electricity itself, a vesicle, a cell, a little boat, as it were, is formed, which floats it to the spot, delivers its charge, and bursts and dies, its duty done, the object of its creation having been performed;—an apt type of the whole and living man, who, when the great object of his creation is performed on earth, himself passes away in death; and happy indeed would he be, were that work so fully, so well, and so invariably done. These little wrecked, these bursted boats, have been collected, and ascertained to be made invariably and almost wholly of two materials—phosphorus and lime, which also are constituents of the brain itself. This phosphorus and lime are supplied by what we eat and drink. If we do not eat and drink enough, or if what we do eat and drink has not enough of these constituents; or if, again, it is not perfectly digested, then there is not enough of these constituents to make the necessary boats to freight the nutrient particles to their destination; hence, the man wastes away to skin and bone, and dies—not because he does not eat, but because what he does eat does him little or no good. Especially thus is it in Consumption; a man dies of inanition, or, as physicians say, *an error of nutrition.*

Consumptive people die for want of strength, want of flesh, want of nutriment; not for want of lung substance, as is almost universally supposed. They die, in almost every instance, long before the lungs are consumed, so far as to be incapable of sustaining life. Numerous cases are given where men have lived for years with an amount of available lungs not equal to one-fourth of the whole. They were there, perhaps, but not available, not efficient. The majority of persons who die of Consumption, perish before a *third* of the lungs have consumed away, in consequence of loose bowels, torpid liver, indigestion, night sweats, want of sleep, clogging up of the lungs with matter and mucus by the daily use of cough drops, balsams, tonics, or other destructive agents. These symptoms need but be controlled to protect life indefinitely; that is to say, if the symptoms were prescribed for according to general principles, and properly nursed, letting the Consumptive portion of the disease alone, it would sometimes cure itself, or at least allow the patient to live in reasonable comfort for a number of years.

The reader may almost imagine that he has a clue to the cure of Consumption, if he could but give the patient phosphorus and lime, or phosphate of lime—that is, burnt bones—eight or ten grains, with the first mouthful of each meal, so as to let it be mixed with the food and carried with it into the blood; from twenty to thirty grains being daily needed in health. The scientific world were charmed less than a hundred years ago by the discovery of oxygen. It was supposed that as oxygen was the constituent of the air which imparted vitality to the blood, gave it its purity, its activity, and filled the man with life and animation, nothing was needed but to take enough oxygen to purify the blood, and thus strike at the root of all disease. Accordingly, the oxygen was prepared and administered. The recipient revived, was transported, was fleet as the antelope, could run with the wind. He smiled, he fairly yelled for joy, and—died, laughing, or from over excitement. The machine worked too fast; it could not be stopped, and pure oxygen has never been taken for health since.

Thus it will, perhaps, always be with artificial remedies; they cannot equal those which are prepared in Nature's manufactory. The phosphate of lime, in order to answer the purposes of nature, must be eliminated from the healthful digestion of substantial food in the stomach, and the only natural and efficient means of obtaining the requisite amount is, to regulate the great glands of the system in such a manner as to cause the perfect digestion of a sufficient amount of suitable food, ☞ and this is within the power of the scientific practitioner, in the great majority of cases of Consumption, when attempted in its early stages; but for confirmed Consumption—that is, when the lungs have begun to decay away, it is criminal to hold out any promises of cure, or even of essential relief, in any given instance.

It is often stated as disparaging to physicians, that, notwithstanding the general increase in knowledge, in all departments, and the claim that medicine is reduced almost to a science, that human life is gradually shortening. There is great reason why men should not live so long as formerly. As a nation, we live more luxuriously; our habits of eating and sleeping have become more artificial, more irregular. Large numbers of people have no regular occupation. Our young women are trained in female boarding schools, which, with rare exceptions, are academies of mental, moral, and physical depravation; where novel reading in secret, and a smattering of everything in public, with a thorough practical knowledge of nothing, is the order of the day. From graduation to marriage nothing is done to establish the constitution, to make firm the health—no instructions given as to how that health may be preserved, no active teaching as to household duties, no invigorating morning walks, no wholesome, elegant, and graceful exercises on horseback. The days are spent in eating, in easy lounging, in ceremonial visitings, in luxurious dreaminess over sentimental fictions; their nights in heated rooms or crowded assemblies of hot and poisoned, if not putrid air. No wonder that with educations like these, the girls of our cities and larger towns fade away into the grave long before they reach the maturity of womanhood.

Our young men, also, in cities and large towns especially, grow up in too many instances without any stamina of constitution. Bad practices—drinking, chewing, smoking, theatre going, secret society gatherings—involving late hours, late suppers, late exposures, private indulgences—these destroy the health, deprave the morals, and waste the energies of the whole man. Many are permitted to grow up without any trade, trusting to a wealthy parentage, or political influence, or the name of a profession, entered only for show and not for practical life. Others grow up as clerks in stores, banks, offices, with good salaries it may be; but when the merchant has become a bankrupt, the offices failed, the banks broken, the party in power defeated, their occupation is gone, their resources are exhausted; they lounge about waiting for a place, the clothes are wearing out, the board bill is in arrears, independence lost, spirits broken, mind irritated, disposition soured, and the first crime is committed—that of engaging board without any certain means of paying, or leaving a struggling widow in arrears;—the proud, the high-minded, the well-dressed, courteous, and cheerful-faced young man of six months ago has made his first step towards degradation, by making a toiling woman give him for nothing the bread and meat which she had earned in toil and sweat, and tears perhaps, and which the children of her own bosom needed. When the honor is lost, low habits and loss of health and life soon follow. Let every young man from the country hesitate to come to the city to try his fortune, unless he have *learned well* an honest and substantial trade; then he may work his way sternly and steadily to usefulness, influence, and wealth It is for want of a suitable education and occupation that such numbers of our young go down to a premature, if not dishonored, grave. But notwithstanding these errors as to the education and employment of our young men and young women, medical writers have been extensively disseminating useful knowledge by means of books, pamphlets, lectures, newspaper articles and the like, in reference to the preservation of health in the nursery, the school-house, the academy, the college—in factories, work-houses, penitentiaries, as to diet, exercise, ventilation, drains, sewerages, house-building; and the general result is, that within three hundred years past, the average length of human life has been increasing and not diminishing. The average age increased two and a half years for the twenty years ending 1820 in the United States. For the fifty years ending in 1831 in France, it increased from 28¾ years to 31¼, notwithstanding the devastations of the wars of Napoleon and the French Revolution. In London, for the century ending 1828, the average age of all who died had increased 4¾ years. In Geneva, 300 years ago, it was 21 years; it is now 41.

Europe is computed to have a population of wo hundred and thirty millions. Not a hundred years ago, Gibbon, the great historian, estimated it at less than one-half. This immense increase has taken place notwithstanding the millions who have emigrated to this and other countries—notwithstanding, too, the far greater drawback, that during a considerable portion of the time the most desolating wars were waged that were ever carried on there.This can only be accounted for by the reforms which medical science has introduced, and the more general diffusion of practical knowledge as to the preservation and promotion of health, in publications made by eminent physicians and surgeons.

As, therefore, a higher degree of medical intelligence has extended the average of human life—in some places fifty per cent., taking all diseases together—it is reasonable to suppose that increased intelligence as to one class of diseases would, in the course of time, have a like happy effect; that if more truthful views as to the nature, *causes*, and symptoms of diseases of the lungs were extensively promulgated among the people, their fearful ravages would be diminished in corresponding proportion.

In 1851, the deaths in Boston, from Consumption alone, were about thirty per cent. of the entire mortality, and the Medical Association announces that it "is steadily on the increase from year to year." If this is the case in Boston, where such large quantities of cod-liver oil have been purely made, and hence more easily and cheaply obtained, it presents a striking and practical contradiction of its curative powers in Consumption, and calls upon us in louder and louder tones to look less to the cure of this terrible scourge, and more to the detection of its early symptoms and its prevention, by scattering intelligence to every family, and on the wings of every wind, as to what are its causes and what these early symptoms are. Such is the object of this publication.

*Patent Medicines* are those whose contents are not made known. A physician who has any respect for himself would scarcely use them, or advise their use. It is a universal custom among all honorable practitioners, to communicate to their brethren any valuable discovery: thus, any one of them is benefited by the discoveries of all the others: they hold their knowledge in common. A remedy discovered to be truly valuable in New York to-day, in the cure of any disease whatever, is, in a few months, known wherever the English language is read and spoken. Thus thousands, scattered over the world, whom the discoverer never could see, are benefited and blessed by his discovery, through the regular practitioner. Some other person obtains this knowledge, prepares the ingredients, disguises them with some inert substance, and sells it as a secret remedy, leaving those to die, as far as he cares, who do not buy from him or his agents; while thousands of others, in other states and countries, perish for the want of a knowledge locked up in his bosom. Any patent medicine is a cure for a given disease, or it is not. If it is not a cure, it is false and criminal to sell it as a cure. If, on the other hand, it is what it professes to be, it cannot be much better than murder to withhold it from those who cannot purchase it, and to allow thousands, at a distance, to die from the want of it, who never heard of it, or, if they did, live too far away to send for it in time. Let those who purchase these articles think of the argument, and aid and abet no more, by their patronage, those who allow their fellow-creatures to die by thousands every year, who would be saved (if what is said be true) by the knowledge of the remedy whose composition is so carefully concealed.

Many things have been passed over in the foregoing pages, which might satisfy the curiosity or interest a large class of readers, but it is not necessary that they should be known, and if known, might have an injurious effect, considering the present state of knowledge on the subject of Consumptive disease; such, for example, as stating what symptoms are infallibly fatal, what kind of persons, as to sex, temperament, color of hair, eyes, skin, make of body, are most liable to it, or having it, have less hope of recovery. For similar reasons, I have given but few fatal cases and their symptoms; for persons having one or more of these same symptoms might conclude that they, too, must die, when those same symptoms, in combination with others, would indicate a very different result. I do not wish the reader to suppose that I do not lose any cases—that few or none die in my hands. I lose patients as other physicians do. I have lost some whom I expected would recover. Nor do I wish to make the impression, that it is a frequent occurrence that persons in the advanced stages of Consumption are restored to comparative health; for it is not a frequent occurrence—it is a rare thing. My object is, first, to show what the early symptoms are; and, second, to induce the reader to make application to me at this early stage, with the full assurance of my belief, that thus one person would not die of disease of the throat or lungs where one hundred now do. In truth, I had greatly rather that persons in the advanced stages would not apply to me; for it at once involves a degree of responsibility and solicitude, which is to extend through weeks and months, and for which any money paid is not the shadow of a remuneration.

I greatly desire it to be understood that I have no magical means of cure. Ailments of the throat and lungs are not to be removed by a box of pills or a bottle of balsam. It is not the work of a day, nor of a week. These cases often require weeks and months of treatment, and of a treatment constantly varying, to meet the varying phases of the disease. Sometimes it occurs, but not often, that a person writes for advice in full, and it is given, and the single prescription, PERSEVERED IN, has effected a happy cure, and months and years after, such persons have come to see me, to express their gratification. At other times, prescriptions are sent, and the persons never heard of afterwards. In nearly all cases, these are young people, or persons who have no energy of character, no perseverance, no determination. For a few days or a fortnight, they give a general attention to the directions, and because they are not cured, break off and apply to some other physician, to follow the same course, or become negligent of themselves, and eventually die. It is a most hopeless task to attempt to cure any of Throat-ail or Consumption who have no energy of character. It is time, and trouble, and money lost, as they are not diseases to be eradicated in a day, by a drop or a pill. It is to be accomplished, if at all, by a determined, thorough and persevering attention, for weeks and sometimes many months, to rational means, ☞ calculated to build up the constitution, with a decreasing use of medicine and an increasing attention to habits of life.

ASTHMA.—I have said but little of this distressing disease. It is not often critical or dangerous until advanced life. As a general rule, it is incurable. Children who have it, sometimes *grow out of it*. In some women, it often disappears at the turn of life; in others, during the years of child-bearing. A *fit of asthma*, as it is called, generally cures itself, by being let alone. An attack is often hastened away by judicious means. In persons of a feeble constitution, it is liable to come or go any day or hour, and prove fatal in marked changes of weather—that is, to very cold, or from cold to a warm, heavy, thawy, foggy atmosphere. The only proper and efficient method of treatment is, to *prevent the attack*, which can be done in the great majority of cases, and for an indefinite length of time. The distinguishing symptom is *want of breath*; the patient feels sometimes as if it would almost kill him to speak two or three words; the necessity of breath is so great, he cannot find time to cough, and represses it, lest it should *take his breath away*. He can neither cough, sneeze, spit, nor speak freely. He sits up, wheezes, throws his head back, wants the doors and windows opened. The attacks generally come on towards the close of the day, and pass off about midnight or soon after, when the cough becomes loose, and large quantities of a substance more or less yellow, pearly, and tenacious, are expectorated; urination becomes copious, and the patient recovers, to be attacked in the same way night after night, until the violence of the disease is expended, and recovery takes place; or if these ameliorations do not occur about midnight, the case is aggravated, and the patient dies in a few hours. This disease is treated more at length in the large edition. It is certain, that in a vast number of cases, whether hereditary or accidental, the attacks can be indefinitely warded off by proper care and habits of life, if the constitution is not much broken.

### CROUP OF CHILDREN.

Many a lovely child is destroyed in a single night by this alarming disease. Its nature is described in the First Part. It is a disease of the windpipe, which is filled or lined with a phlegm, which becomes more and more tough, almost leathery—thickens, and at length closes up the passage to the lungs, and the child dies.

It usually comes on in the night. The distinguishing symptom is a wheezing, barking cough. A mother who has ever heard it once, needs no description to enable her to recognise it again. The first born are most likely to perish with it; simply because the parent has no experience of its nature, and hence is not alarmed in time, or knows not what to do, while the physician is being sent for. In the hope of being instrumental in saving some little sufferer, whose life is inexpressibly dear, at least to one or two, I will make some suggestions, not for the cure of the patient, but to save time. The instant you perceive that the child has *Croup*, indicated by the *barking Cough*, *uneasy breathing*, *restlessness*, send for a physician, and as instantly wrap a hot flannel around each foot, to keep it warm; but while the flannels are being heated, dip another flannel, of two or more thicknesses, in spirits of *turpentine*, or spirits of *hartshorn*; or have a large mustard plaster applied, one that will reach from the top of the throat down to some two inches below the collar bones, wide enough at top to reach half-way round the neck on either side, and nearly across the whole breast at bottom. But it will take time to send for a physician, to prepare flannels, and to make the plaster or obtain the turpentined flannel, and in some cases fifteen minutes is an age—is death, if lost; therefore, while these things are preparing, give the child, if one year old or over (and half as much, if less), about half a teaspoon-ful of Hive Syrup, and double the dose every fifteen minutes until vomiting is produced; and every half hour after vomiting, give half as much as caused the vomiting, until the physician comes, or the child ceases to cough, when he breathes free, and is safe. If you have no Hive Syrup, give a teaspoon-ful of Syrup of Ipecac, and double the dose every fifteen minutes until vomiting is produced. If you have been so thoughtless as to have nothing at all, boil some water, keep it boiling, dip a woolen flannel of several folds into it, squeeze it out moderately with *our hand*, and apply it as hot as the child can possibly bear to the throat, and in from one to three minutes, according to the violence of the symptoms, have another to put on the instant the first is removed, and keep this up until the breathing is easy and the cough is loose and the phlegm is freely discharged, or until the arrival of the physician.

I wish to impress upon the reader's mind a few disconnected subjects. Consumption most generally comes on by a slight cough in the morning, about the time of rising or first stirring about. The existence of tubercles in the lungs is not necessarily fatal; they remain dormant for a life-time, unless irritation or inflammatory action is excited by *bad colds neglected*, or *exhausting habits or diseases, or debilitating occurrences, or wasting indulgences*. These things throw more persons into fatal Consumption than are destroyed by the hereditary form of the disease; and these should be, as they can in very many instances, safely remedied.

The following recipes are frequently referred to:—

*How to Toast Bread.*—Keep the bread a proper distance from the fire, so as to make it of a straw color. It is spoiled if it is black, or even brown.

*Toast Water.*—Take a slice of bread about three inches across and four long, a day or two old. When it is browned, not blackened, pour on it a quart of water which has been boiled and afterwards cooled. Cover the vessel, and after two hours, pour off the water from the bread gently. An agreeable flavor may be imparted by putting a piece of orange or lemon peel on the bread at the time the water is first poured on the bread.

*Barley Water.*—Take two tablespoons of pearl barley, wash it well in cold water, then pour on it half a pint of water, and boil it fifteen minutes; throw this water away, then pour on two quarts of boiling water, and boil down to a pint; then strain it for use. An ounce of gum arabic dissolved in a pint of barley water is a good demulcent drink.

*Flax-seed Tea.*—Take an ounce or full table-spoon of flax seed, but not bruised, to which may be added two drams of bruised liquorice root; pour on a pint of boiling water, place it covered near the fire for four hours, strain through a cotton or linen rag. Make it fresh daily.

*Tamarind Whey.*—Two tablespoon-fuls of tamarind, stirred in a pint of boiling milk; then boil for fifteen minutes, and strain.

*Wine Whey.*—Take a pint of milk, put it on the fire; as soon as it begins to boil, pour on eight or ten tablespoons of Madeira wine, in which has been stirred two teaspoons of brown sugar; stir the whole until it has been boiling for fifteen minutes; then strain through a cloth.

*Boiled Flour and Milk.*—Take a pint of flour; make it into a dough ball with water; tie it tightly in a linen bag; put it into a pan of water, covering the ball, and let it boil ten hours; place it before the fire to dry, cloth and all; take it out of the cloth, remove the skin, dry the ball itself. Grate a tablespoon of this, and stir it into a pint of boiling milk, until a kind of mush is formed.

*Boiled Turnips.*—Small turnips boiled make one of the best articles of food which invalids and convalescents can use. Carrots may be added; half and half. Boil them once; repeat the boiling in fresh water until they are quite soft; press the water out through a coarse cloth; then mix enough new milk to form a kind of pulp; season with salt, and then place them before the fire until it is a little dry or crusted.

*Beef Tea.*—Cut into thin slices a pound of lean meat, pour on a full quart of cold water, let it gradually warm over a gentle fire; let it simmer half an hour, taking off the skum; strain it through a napkin. Let it stand ten minutes, then pour off the clear tea.

*Cracked Wheat.*—Dry some common wheat, then grind it in a coffee mill; boil it three or four hours; add a little salt, a little milk, butter, cream, or molasses may be added, as in using homminy. It should be always washed clean, and then boiled long enough to become of the consistence of boiled rice or hominy. A pint of wheat dried and grounnd is enough for a day; not to be used for supper.

*Dandelion Diet Drink.*—Take three ounces of the bruised root of the dandelion flower, which should be gathered in July, August, and September; pour on a quart of water, boil it to a pint, and strain it.

| | | |
|---|---|---|
| 60 Drops | make | one Teaspoon. |
| 4 Teaspoons | " | one Tablespoon. |
| 2 Tablespoons | " | one Ounce. |
| 2 Ounces | " | one Wine-glass. |
| 2 Wine-glasses | " | one Gill or Teacup. |
| 4 Gills | " | one Pint. |

I greatly desire that nothing I have written should excite unreasonable expectations as to the speediness of cure of the diseases treated of; they come on slowly, are sometimes for years gathering force in the system, and hence it is unreasonable to suppose that they are to be eradicated except by energetic treatment, long-continued, unless attended to in their very first stages. The patient, page 107 top of second column, expressed himself as being cured in two days:—it was three months before every remnant of disease seemed to have left his throat. Remember this, if no other sentence—attend at once to the first morning cough, or frequent hawking, hemming, swallowing, or want of clearness of voice of two weeks' continuance; otherwise, in nine cases out of ten, a fatal Consumption will be the result.

The charge for answering a letter desiring an opinion of a case, is Five Dollars; and Ten Dollars for a personal examination and opinion. Advice is given by letter or at the office personly, according to the nature of the case and the circumstances of the patient; all charges must be paid at the time of consultation. The descriptions given must include an answer to the following questions:

Are you easily chilled? Do you take cold readily? Are you inclined to be thirsty, forenoons? —Are you troubled with cold feet or hands? Is there shortness of breath in walking briskly, especially on rising ground, or up stairs? Your best weight; usual; present? Do you perspire readily? Have you any discomfort after meals? Any bad taste in the mouth of mornings? Do the bowels act regularly every day? Are you regular otherwise? Do you live in town or country? What is your age, height, occupation? Are you married, and have you children? How often does your pulse beat in a minute when you are at rest, about the middle of the A. M. or P. M.? Is your voice natural? Have you reason to believe that any of your symptoms are hereditary? The above statements are made and questions asked, to save time which, in some cases, makes all the difference between life and death.

Address Dr. W. W. Hall, 2 West 43d St., N. Y.

